# Assessing COVID-19–Related Knowledge, Attitudes, and Practices Among Hispanic Primary Care Patients: Protocol for a Cross-sectional Survey Study

**DOI:** 10.2196/25265

**Published:** 2021-01-27

**Authors:** Zoe C Burger, Shivani N Mehta, Dayanna Ortiz, Sekboppa Sor, Jigna Kothari, Yvonne Lam, Meena Meka, Ajay Meka, Timothy Rodwell

**Affiliations:** 1 School of Medicine University of California San Diego La Jolla, CA United States; 2 University of San Diego Department of Global Health La Jolla, CA United States; 3 Amistad Medical Clinic Santa Ana, CA United States

**Keywords:** COVID-19, knowledge, attitude, practices, Hispanic, California, protocol, cross-sectional, survey

## Abstract

**Background:**

Recent epidemiological data indicate that minority groups, especially Hispanic communities, experience higher rates of infection, hospitalization, and death due to COVID-19. It is important to understand the nature of this health disparity and the socioeconomic or behavioral factors that are placing Hispanic communities and other minority populations at higher risk for morbidity and mortality.

**Objective:**

The purpose of this project is to assess current COVID-19–related knowledge, attitudes, and practices (KAP) among a predominantly Hispanic population from Orange County, California, and identify risk factors that may contribute to increased susceptibility and vulnerability to contracting SARS-CoV-2.

**Methods:**

Our Orange County–wide community survey consists of quantitative survey questions in four domains: demographic information, COVID-19 knowledge questions, COVID-19 attitude questions, and COVID-19 practices questions. The survey questions are adapted from recent global KAP studies. Participants are being recruited from Amistad Medical Clinic, a private primary health clinic group in Orange County that treats a predominantly Hispanic population. Patients recruited during telehealth visits are surveyed remotely by telephone, and those recruited during in-person clinic visits are surveyed in person. Surveys are conducted by trained members of the study team who are native to the community setting.

**Results:**

As of October 12, 2020, we had recruited and enrolled 327 participants. Data collection occurred June 26th to October 30th. Data analysis is ongoing.

**Conclusions:**

Very few current COVID-19 studies focus on the perspective and experience of minority populations. Because Hispanic communities are disproportionately affected by COVID-19, it is important to understand the factors the contribute to this disparity and the next steps that should be taken to reduce the COVID-19 burden in this population. We believe that our study model of partnering with a local clinic system that serves our study population can be expanded to other settings to compare COVID-19 KAP and associated factors within different minority communities.

**International Registered Report Identifier (IRRID):**

DERR1-10.2196/25265

## Introduction

The COVID-19 pandemic continues to grow in scale and scope globally, and it represents a major threat to the health and safety of communities worldwide. However, recent epidemiological data indicate that minority populations in the United States, especially Hispanic communities, are disproportionately infected and hospitalized with COVID-19 [1-4]. To develop better public health and preventative measures, it is critical that we collect local data to understand the potential risk factors and perspectives of these populations as they experience the pandemic. This information is necessary to assess existing measures of prevention and create novel, targeted strategies to reduce the burden of COVID-19 on minority populations and control the spread of the virus in all communities [5].

As of October 2020, SARS-CoV-2 has infected more than 7 million people and has resulted in more than 200,000 deaths in the United States [6]. Globally, cases in the United States currently account for over 25% of COVID-19 cases worldwide, and the number of cases is continuing to grow at a concerning rate [7].

This pandemic has highlighted the health disparities that exist in our country, and some of the most apparent inequities are seen among the Hispanic population. In California, Hispanic people comprise only 38.9% of the population; however, they account for 56.1% of COVID-19 cases and 45.6% of COVID-19–related deaths in the state [8]. States across the country report similar case rate disparities, such as Oregon (13% of population vs 39.8% of COVID-19 cases), Washington (13% of population vs 44% of COVID-19 cases) and Utah (14% of population vs 39.4% of COVID-19 cases) [1]. In Chicago, Illinois, COVID-19 cases among Hispanic patients represent almost half of the total cases in the city [9].

Many hypotheses have been proposed for why minority populations and Hispanic communities are disproportionately affected by COVID-19, including limited access to care, increased exposure, and higher prevalence of comorbidities leading to worse outcomes [10,11]. First, it is important to note that Hispanic individuals have the highest uninsured rates of any racial or ethnic group within the United States, creating significant barriers to accessing COVID-19 testing and health care [12]. Additionally, individuals in Hispanic communities are more likely to be employed by public-facing businesses and institutions, including the food service, transportation, and sanitation industries [13]. Because these individuals do not enjoy the privilege of working from home and were required to come into work during stay-at-home orders, they would have been disproportionately exposed to the virus. Living conditions may also play a role in the increased exposure of Hispanic populations to the novel virus; many Hispanic families live in densely populated areas and multigenerational households, which can facilitate person-to-person transmission [10]. Finally, the Hispanic population has an increased burden of comorbidities, including diabetes, hypertension, and heart disease [14]. While these comorbidities do not increase a person’s susceptibility to contracting the virus, they lead to worse health care outcomes. In a recent study of the disproportionate rise in COVID-19 cases among our study population in Orange County, California, researchers at the University of California, Irvine suggested that low income level, household density, lower educational attainment and lower health care coverage are risk factors for COVID-19 infection [15].

While research and epidemiological data have identified COVID-19–related health inequities among minority populations, few efforts have been made to understand how these populations cope with and conceptualize the pandemic. One survey conducted by the Pew Research Center found that Hispanic individuals are more likely to be concerned about COVID-19 compared to the general public. Approximately 65% of Hispanic adults say that the novel virus is a major threat to the health of the US population as a whole, compared with approximately half (47%) of the general public. Additionally, Hispanic adults were more likely to express concern that they would be hospitalized with COVID-19 or spread the virus unknowingly to others [16,17]. These concerns warrant further exploration, especially in the context of a vaccine being released in the coming months. A recent poll conducted by the Associated Press-NORC Center for Public Affairs Research found that only 25% of African Americans and 37% of Hispanic individuals would get a COVID-19 vaccine, compared to 56% of whites [18]. Understanding the attitudes and perspectives of Hispanic communities as they experience the COVID-19 pandemic is equally significant to develop effective, culturally specific public health policies and messaging.

A knowledge, attitudes, and practices (KAP) survey is an ideal tool to understand the risk factors that could predispose Hispanic communities to excess COVID-19 infection and mortality. The data from our survey have yet to be analyzed; however, we believe that understanding these risk factors, as well as the perspectives of this population, will enable public health and clinical institutions to implement effective and culturally relevant interventions to reduce these outcomes. We believe that the methodology of our study to collect KAP and demographic information using a clinic telehealth setup during the COVID-19 pandemic can be an extremely valuable method that can be used to recruit participants in future studies.

## Methods

### Settings and Population

Our quantitative surveys are being conducted at two Amistad Medical Clinic (AMC) health care centers in Orange County, California: AMC Santa Ana and AMC Anaheim. Both clinics are located in predominantly Hispanic areas of Orange County and serve as private primary care clinics for community members. Approximately 90% of the patients served by these clinics identify as Hispanic. The clinics accept California state, federal, and private insurance; therefore, they are accessible to all community members. The AMC centers serve approximately 0.5% of the populations of Santa Ana and Anaheim, respectively.

### Questionnaire

Our survey consists of four question domains with a total of 33 questions, some of which have been adapted from previous COVID-19–related KAP studies [19-21]. The first portion of the survey is focused on sociodemographic data, including age, gender, education, marital status, race, ethnicity, and employment status. We also inquire if the California stay-at-home order has impacted the participant’s employment and whether the participant has ever tested positive for COVID-19. Most importantly, we ask if they were required to leave their home to work during the stay-at-home order, placing them at risk of contracting the disease.

The second portion of the survey is used to measure COVID-19–related knowledge. The knowledge questions are a series of 1 multiple choice question, 1 open-ended question, and 7 “yes” or “no” questions. These questions assess the patients’ understanding about disease symptoms, transmission, spread, and susceptibility.

The third section of the survey is used to measure COVID-19–related attitudes. The attitudes portion of the survey consists of 5 questions that are answered using a 5-point Likert scale and 2 multiple choice questions. This portion of the survey is used to measure participants’ general perceptions about COVID-19 regarding (1) governments’ effectiveness in decreasing disease prevalence; (2) emotions during the pandemic, including anxiety, fear, anger, and optimism; and perceived risk of contracting COVID-19.

The final portion of the survey consists of COVID-19–related practices. The practices questions consist of 2 “yes” or “no” questions and 2 questions using a 5-point Likert scale. Our questions help us discern the steps that our participants are taking to protect themselves and the community from viral spread, and this portion includes questions regarding (1) attending gatherings and entering public spaces; (2) washing hands frequently; (3) wearing masks outside; and (4) interest in and concerns about COVID-19 testing and future vaccines.

Our full questionnaire is provided in Multimedia Appendix 1.

### Recruitment

We have integrated our study within the majority of telehealth appointments in the clinics and in a few in-person appointment systems.

After the patient has completed their telehealth appointment, they are invited to participate in the survey study by their primary care physician, who then documents the patient’s willingness to participate by name, number, and availability on a secure drive. The drive is then accessed by the study interview team, and a member of the team calls the patient within 48 hours of recruitment. The interview team member obtains verbal consent during the telephone call and then proceeds to ask the survey questions.

In-person appointments follow a similar process. After the provider recruits a willing volunteer, a member of the interview team who is present at the clinic obtains verbal consent in person and then proceeds to ask the survey questions.

### Sample Method and Sample Size

Our survey uses a consecutive sampling method. The primary care physicians attempt to recruit each of their patients after their telehealth or in-person appointment. If the patient agrees to participate in the study, they are contacted by the study interview team to undergo the verbal consent process and complete the survey. Our sample size will reflect the number of people who are recruited and surveyed. We aim to interview approximately 400 participants over a span of 3 months.

### Results

The first study patient was enrolled on June 26, 2020, and recruitment and enrollment proceeded until October 30th. As of October 12, 2020, we had recruited and enrolled 327 participants, and we projected that 400 participants would be recruited and enrolled by the end of the recruitment period. We will conduct data analysis after the recruitment period on October 30, 2020.

The study was approved exempt from review by the University of California, San Diego Institutional Review Board on June 22, 2020.

## Discussion

Hispanic and other minority communities are experiencing disproportionately high rates of COVID-19 infection and severe outcomes in the United States. In Orange County, California, Hispanic individuals represent 35% of the population but account for almost half of COVID-19 infections and deaths [22,23]. Therefore, it is important to understand and assess the nature of this increased risk in Hispanic communities to contribute to future disease prevention in the next stages of this pandemic.

The KAP survey format has historically been a useful tool in identifying risk factors that exist within a specific population regarding a highly prevalent disease [24]. In addition, the KAP survey provides insight into the perceptions and stigmas that may be preventing people from seeking health care and following safety guidelines when experiencing a pandemic. Finally, some KAP surveys can also identify behavioral risks that cause a population to be more susceptible to a disease [19,25]. For example, our survey will identify individuals who worked outside of their home during the stay-at-home order because they were considered to work in “essential” industries.

We hope that the hybrid remote/in-person design of our pilot study can be used as a foundation for future COVID-19–related KAP studies. Specifically, we believe that the data from our study will pave the way for future COVID-19–related KAP studies in minority populations. Data from future KAP surveys will be used to create effective, evidence-based public health measures and highlight specific risk factors that affect vulnerable minority communities.

Our method of recruitment and data collection is novel and extremely useful for conducting cross-sectional surveys during the COVID-19 pandemic, where most medical practitioners are relying on telehealth appointments [26]. It is critical that during the pandemic, we are able to safely conduct scientific studies without compromising the health of the participants and the study team or the accuracy of the data.

Most importantly, we believe that future COVID-19–related KAP studies can partner with local primary care clinics because these clinics have maintained longevity, rapport, and trust with their patients. Therefore, patients are more inclined to be candid when answering surveys, heavily decreasing response bias. Private primary care clinics within minority communities are also agents for public health changes on a local level [27,28]. The clinic staff are typically advocates of their communities and have autonomy over their practices; hence, they can be open to taking study conclusions and directly implementing them in their patient care routine (ie, increasing COVID-19 education for their patients to prevent future misconceptions).

Our cross-sectional pilot study evaluates the COVID-19–related knowledge, attitudes, and practices of the Hispanic adult population to inform the development of evidence-based COVID-19 prevention policies and public health measures. It is important to partner with local clinics that have existing infrastructures and relationships with their communities to study and ultimately help these vulnerable populations in the era of COVID-19.

## Appendix

Multimedia Appendix 1 Study questionnaire.

## Abbreviations

KAP: Knowledge, Attitudes, and Practices
AMC: Amistad Medical Clinic
