# Supplementary material for: Assessing COVID-19–Related Knowledge, Attitudes, and Practices Among Hispanic Primary Care Patients: Protocol for a Cross-sectional Survey Study
Source: JMIR Res Protoc. 2021 Jan 27;10(1):e25265. doi: 10.2196/25265 (PMC7842854; doi:10.2196/25265)
Supplement: Multimedia Appendix 1 [file resprot_v10i1e25265_app1.pdf]

SECTION 1: SOCIO-DEMOGRAPHIC

1) How old are you? ______

2) What is your gender?
   a) Female
   b) Male
   c) Self-identify
   d) Don’t Know
   e) Refuse to Answer

3) What is your marital status?
   a) Single, Never Married
   b) Married
   c) Divorced
   d) Separated
   e) Widowed
   f) Don’t Know
   g) Refuse to Answer

4) What is your highest level of education?
   a) Elementary School
   b) Middle School
   c) High School
   d) College Degree

By Zoe Burger and Shivani Mehta
UC San Diego School of Medicine
6/14/2020

e) Graduate School

f) Don't Know

g) Refuse to Answer

5) Do you identify as Hispanic?

a) Yes

b) No

c) Don't Know

d) Refuse to Answer

6) What race do you identify as?

a) White

b) Black or African American

c) American Indian or Alaska Native

d) Asian

e) Native Hawaiian and Pacific Islander

f) Other

g) Don't Know

h) Refuse to Answer

7) Are you currently employed?

a) Yes

b) No

c) Student

d) Retired

e) Refuse to Answer

8) Did the COVID-19 “stay at home” order cause you to lose your job or go on a temporary leave?

a) Yes

b) No

c) Refuse to Answer

d) Not employed before COVID-19 “stay at home” order (March- June)

9) If you are employed, did you have to leave your home to work during the “stay at home” order (March-June)?

a) Yes

b) No

c) Refuse to Answer

d) Not employed

10) What is your annual household income?

a) Less than $25,000

b) $25,000 to $34,999

c) $35,000 to $49,999

d) $50,000 to $74,999

e) $75,000 to $99,999

f) $100,000 to $149,999

g) $150,000 or more

h) Don’t Know

i) Refuse to Answer

11) Have you ever tested positive for COVID-19?

a) Yes

b) No

c) Don’t Know

d) Refuse to Answer

SECTION 2: COVID-19 Knowledge

12) What do you think the main symptoms of COVID-19? (Multiple Mark)

a) Cough

b) Headache

c) Shortness of Breath

d) Body aches

e) Fever

f) Loss of taste

g) Loss of smell

h) Sneezing

i) Fatigue (feeling tired)

j) Chills

k) Rash

l) Nausea

m) Vomiting

n) Diarrhea

o) Other _______

p) Don’t Know

q) Refuse to Answer

13) Can people be infected with COVID-19 when an infected person coughs, sneezes, or speaks?

a) Yes

b) No

c) Don’t Know

d) Refuse to Answer

14) Does COVID-19 spread through touching contaminated surfaces and then touching the eyes or mouth?

a) Yes

b) No

c) Don’t Know

d) Refuse to Answer

15) Is there a cure for COVID-19?

a) Yes
b) No
c) Don't know
d) Refuse to answer

16) Is there a vaccine for COVID-19?

a) Yes
b) No
c) Don't know
d) Refuse to answer

17) How far should you be from another person to be safe from COVID-19? (Open-ended)

a) _______
b) Don't Know
c) Refuse to Answer

18) Do only people 65+ years old get severe COVID-19 infection?

a) Yes
b) No
c) Don't Know
d) Refuse to Answer

19) Are people with pre-existing medical conditions (such as diabetes, hypertension, obesity) more likely to experience serious medical problems from COVID-19?

a) Yes

b) No

c) Don't Know

d) Refuse to Answer

20) Can an infected person who does not have symptoms of COVID-19 infect someone else?

a) Yes

b) No

c) Don't Know

d) Refuse to Answer

SECTION 3: COVID-19 Attitude

21) I think that the government will stop the spread of the virus.

a) Strongly Disagree

b) Disagree

c) Neutral

d) Agree

e) Strongly Agree

f) Refuse to Answer

22) I feel nervous about how COVID-19 will impact my future.

a) Strongly Disagree

b) Disagree

c) Neutral

d) Agree

e) Strongly Agree

f) Refuse to Answer

23) I am feeling angry or frustrated because of COVID-19.

a) Strongly Disagree

b) Disagree

c) Neutral

d) Agree

e) Strongly Agree

f) Refuse to Answer

24) I feel scared to leave my home because of COVID-19.

a) Strongly Disagree

b) Disagree

c) Neutral

d) Agree

e) Strongly Agree

f) Refuse to Answer

25) I feel hopeful about the future.

a) Strongly Disagree

b) Disagree

c) Neutral

d) Agree

e) Strongly Agree

f) Refuse to Answer

26) What do you perceive as your biggest risk for COVID-19?

a) Your Age

b) Your Other Health Problems

c) Your Gender

d) Your Race

e) Going to Work

f) Going to Social Gatherings

g) Household Income Level

h) Daily Errands

27) What do you perceive as your biggest risk for getting hospitalized with COVID-19?

a) Your Age

b) Your Other Health Problems

c) Your Gender

d) Your Race

e) Going to Work

f) Going to Social Gatherings

g) Household Income Level

h) Daily Errands

SECTION 3: COVID-19 Practices

28) Have you been to gatherings or public spaces with more than 10 people in the last week?

a) Yes

b) No

c) Don't Know

d) Refuse to Answer

29) Do you wash your hands when you come into your house from outside?

a) Never

b) Rarely

c) Sometimes

d) Most of the Time

e) Always

30) Do you wear a mask when you go outside your home?

a) Never

b) Rarely

c) Sometimes

d) Most of the Time

e) Always

31) Would you like to be tested for COVID-19 if it was available to you?

a) Yes

b) No

c) Don’t Know

d) Refuse to Answer

32) If a COVID-19 vaccine was available, would you get one?

a) Yes

b) No

c) Maybe

d) Refuse to Answer

33) If not, why would you not get the vaccine?

a) I don't think it would work

b) I'm concerned about the possible side effects

c) I'm worried about the cost/financial reasons

d) Don't think its necessary

e) Other people will get it so I don't need to

f) Refuse to Answer

g) Would take the Vaccine
